# Supplementary figures and images for: Lack of authentic atrial fibrillation in commonly used murine atrial fibrillation models
Source: PLoS One. 2022 Jan 7;17(1):e0256512. doi: 10.1371/journal.pone.0256512 (PMC8741011; doi:10.1371/journal.pone.0256512)

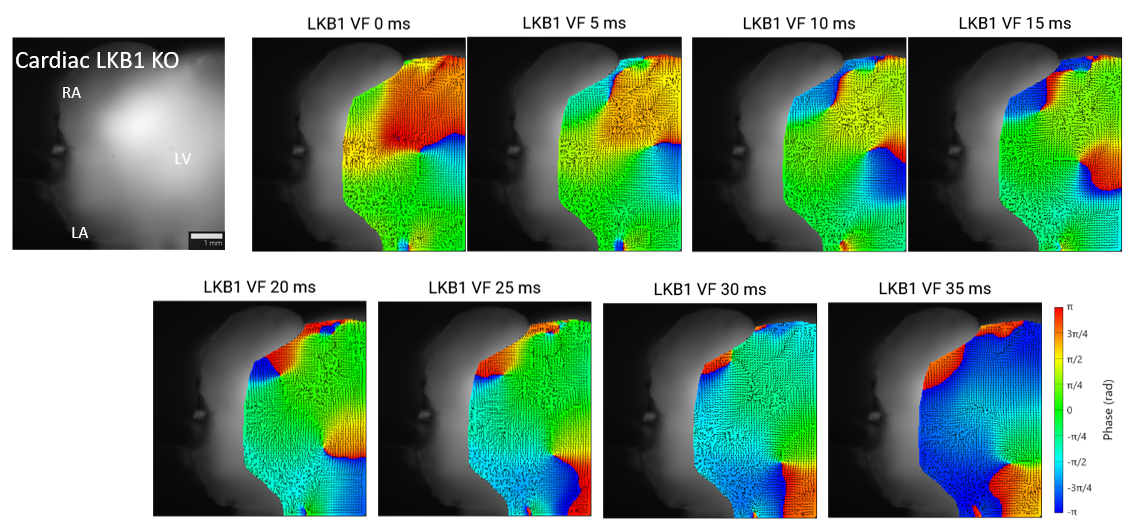

Supplement: S1 Fig — Phase maps of the ventricular having ventricular fibrillation (VF). ~30 Hz of spiral wave was observed. VF was established by several attempts of right ventricular 100 Hz burst pacing stimulations for 5 sec. (TIF) [file pone.0256512.s001.tif]

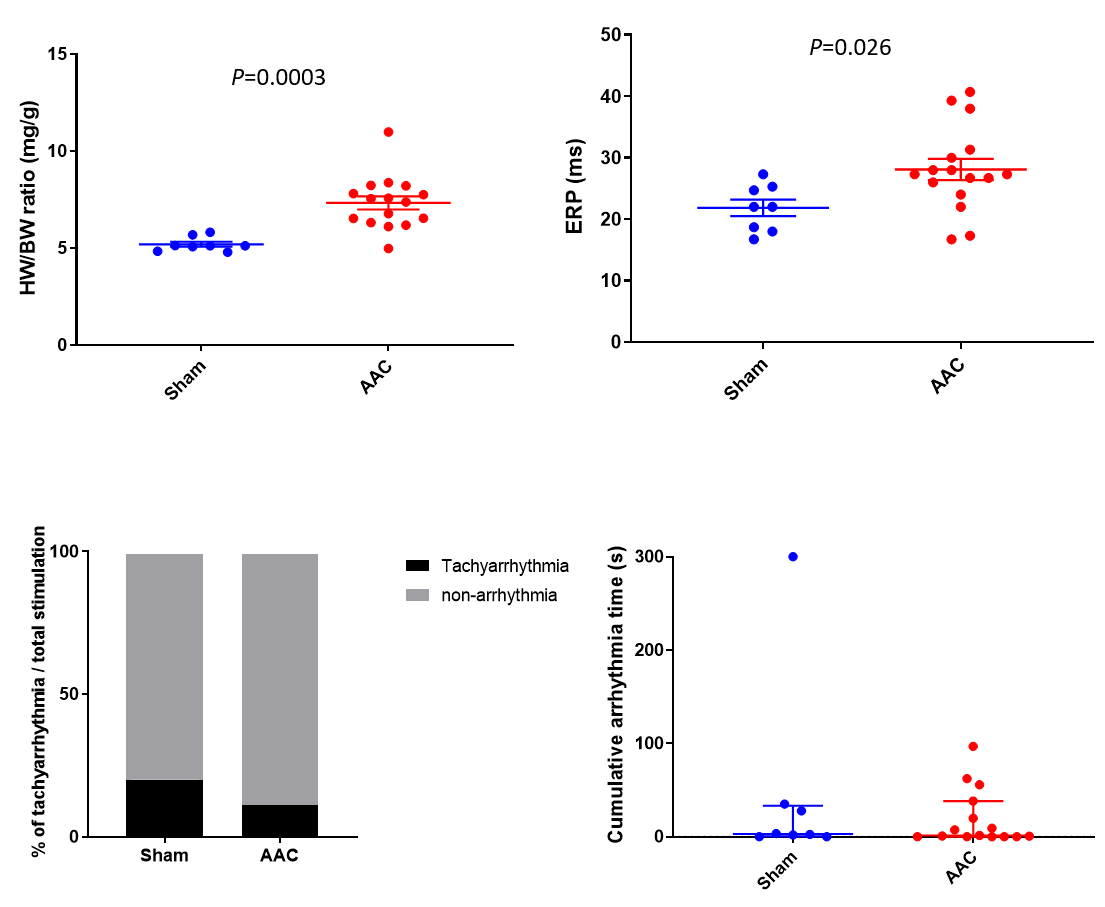

Supplement: S2 Fig — A: Heart weight / body weight ratio in sham (n = 8) and AAC (n = 16) mice at 5 weeks after surgery. B: Right atrial effective refractory period (ERP) in sham and AAC mice. Each point indicates individual animal data, and lines represent mean ± SEM. C: % of positive atrial tachyarrhythmias from total burst pacing stimulations in each group. D: Cumulative tachyarrhythmia time in each animal. Each point indicates individual animal data, and lines represent median ± IQR. (TIF) [file pone.0256512.s002.tif]

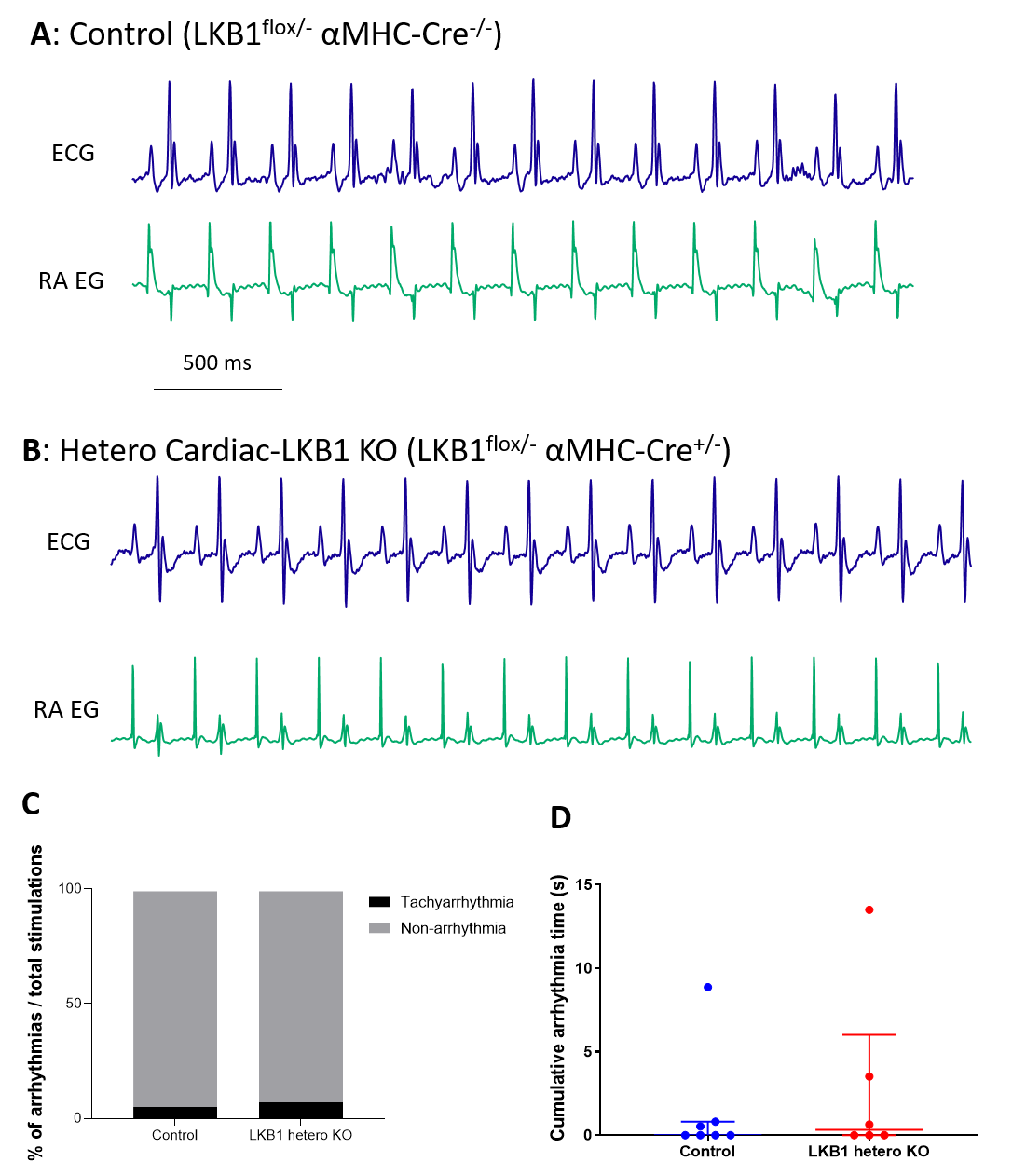

Supplement: S3 Fig — Burst pacing induced tachyarrhythmias in Cardiac-LKB1 heterozygous KO mice. A: Control mouse shows sinus rhythm, clear P wave and corresponding RA (RA) signal on RA EG. B: Cardiac-LKB1 heterozygous KO mouse also shows sinus rhythm. C: % of positive atrial tachyarrhythmias from total burst pacing stimulations in each group. D: Cumulative tachyarrhythmia time in each animal. Each point indicates individual animal data, and lines represent median ± IQR. (TIF) [file pone.0256512.s003.tif]
