# Supplementary material for: Lack of authentic atrial fibrillation in commonly used murine atrial fibrillation models
Source: PLoS One. 2022 Jan 7;17(1):e0256512. doi: 10.1371/journal.pone.0256512 (PMC8741011; doi:10.1371/journal.pone.0256512)
Supplement: S1 Table — Reagents information for mRNA quantifications in the evaluated genes and internal control genes. Since Thermo Fisher Scientific does not disclose primer sequences, assay identification number of each gene are shown. (DOCX) [file pone.0256512.s012.docx]

**S1 Table. Reagents information for qRT-PCR**

| **Gene** | **Assay ID** |
| --- | --- |
| LKB1 | Mm00488476 |
| TGFb1 | Mm00441724 |
| Col1a1 | Mm00801666 |
| CTGF | Mm01192933 |
| SCN5A | Mm01342518 |
| CX40 | Mm00433619 |
| TATA-box binding protein | Mm00437762 |
| Actin | Mm0219580 |
| β-2-microglobulin | Mm01277042 |
